# Mechanical regulation of macrophage function - cyclic tensile force inhibits NLRP3 inflammasome-dependent IL-1β secretion in murine macrophages

**DOI:** 10.1186/s41232-019-0092-2

**Published:** 2019-02-07

**Authors:** Kentaro Maruyama, Eiji Nemoto, Satoru Yamada

**Affiliations:** Department of Periodontology and Endodontology, Tohoku University Graduate School of Dentistry, Sendai, Japan

**Keywords:** Mechanical stress, Cyclic stretch, Inflammasome, Macrophage

## Abstract

Mechanical stress maintains tissue homeostasis by regulating many cellular functions including cell proliferation, differentiation, and inflammation and immune responses. In inflammatory microenvironments, macrophages in mechanosensitive tissues receive mechanical signals that regulate various cellular functions and inflammatory responses. Macrophage function is affected by several types of mechanical stress, but the mechanisms by which mechanical signals influence macrophage function in inflammation, such as the regulation of interleukin-1β by inflammasomes, remain unclear. In this review, we describe the role of mechanical stress in macrophage and monocyte cell function.

## Mechanical stress and tissue homeostasis

Mechanical stress maintains tissue homeostasis by regulating cellular functions such as development, inflammation, bone remodeling, and tumor progression [1–4]. Most cells in connective tissue receive various mechanical stresses such as stretch force, compressive force, shear stress, and hydrostatic pressure [2]. Several tissues, such as the heart, lung, bone, gut, and periodontal ligament, are affected by mechanical stress, and cells in these tissues are involved in tissue homeostasis [5–12]. Integrin, voltage-gated Ca^2+^ channels, and focal adhesion kinase (FAK) are known as mechanosensors that sense mechanical stress in cells. In addition to these sensors, cytoskeleton dynamics affect how cells respond to mechanical force [2]. Mechanical stress activates signaling pathway downstream of mechanosensors, such as the mitogen-activated protein (MAP) kinase pathway or cytoskeletal reorganization, which results in expression of specific genes or post-transcriptional gene regulation [13]. While physiological mechanical stress maintains tissue homeostasis, excessive force or absence of mechanical stress causes various pathological changes such as pro-inflammatory responses and tissue atrophy [14]. For example, excessive mechanical stimuli by mechanical ventilation causes lung inflammation [15]. Mechanical stress from excessive occlusal force or orthodontic tooth movement in periodontal tissue promotes inflammation of periodontal tissue [12, 16]. Low shear stress because of stagnant blood flow in arteries promotes inflammation and is a factor in arteriosclerosis [17]. Loss of occlusal force in periodontal tissues causes atrophy of periodontal ligament tissue and induces interleukin (IL)-1β gene expression [18, 19]. Therefore, appropriate mechanical stress is needed to maintain homeostasis in physiological microenvironments.

## Macrophages

Macrophages have a central role in immune reactions through phagocytosis of pathogenic microorganisms, by releasing inflammatory mediators, such as interleukin, and by inducing inflammation [20]. Macrophages not only eliminate pathogenic bacteria but also maintain tissue homeostasis by removing apoptotic cells and repairing tissue following inflammation [21]. Macrophages already present in specific tissue are called tissue-resident macrophages [22]. Tissues-resident macrophages are derived from the yolk sac at the embryonic stage, are replicated in tissues to maintain cell number, and have different morphology and function depending on the tissue [22]. For example, macrophages in the lung are called alveolar macrophages, those in the liver are called Kupffer cells, and macrophage-like microglia operate in the nervous system. The diversity of tissue-resident macrophages is related to interactions with cells in supporting tissues [22]. However, in the case of tissue injury or infection, monocytes derived from bone marrow circulating in peripheral blood migrate to the affected tissue, differentiate into macrophages, and are involved in the inflammatory response [23]. The cellular functions of tissue-resident macrophages and peripheral blood-derived macrophages are affected by the tissue-specific microenvironment, which can create many types of mechanical stress on cells [24, 25]. Stiffness and topography, which are mechanical properties of the extracellular matrix, regulate the differentiation, proliferation, and function of macrophages. In addition, macrophages present in these tissues are exposed to dynamic mechanical loading, such as stretch and compression, not only continuously but also cyclically. In this review, we describe the role of mechanical loading in macrophage and monocyte cell function.

## Mechanical force and macrophages

It has been reported that mechanical stress, such as stretch and compression, regulate monocyte/macrophage function in terms of cytokine and proteinase expression and cell differentiation as shown in Table 1. Cyclic stretch promotes the secretion of IL-6, IL-8, and tumor necrosis factor (TNF)-α in human alveolar macrophages, human monocyte-derived macrophages, and a human macrophage-like cell line (THP-1) [26]. In THP-1, cyclic stretch induces cyclooxygenase (COX)-2 gene expression, and a combination of cyclic stretch and titanium particles promotes prostaglandin E_2_ (PGE_2_) production [27]. In rat peritoneal macrophages, static stretch induces inflammatory cytokine gene expression such as inducible nitric oxide synthase (iNOS) and IL-6 [28]. On the other hand, there are some reports suggesting that mechanical stress does not particularly affect cytokine production. In rat alveolar macrophages, cyclic stretch does not affect TNF-α and IL-6 secretion [29]. In a mouse macrophage-like cell line (RAW264.7) and bone marrow-derived macrophages, cyclic biaxial stretch does not affect the expression of IL-1β, IL-6, TNF-α, and COX2 [30]. Macrophages are involved in the remodeling of the extracellular matrix via secretion of matrix metalloproteinase (MMP), and cyclic stretch is involved in the regulation of secretion. In human monocyte-derived macrophages, cyclic stretch induces the expression of MMP-1 and MMP-3 [31]. Cyclic stretch also regulates the differentiation of macrophages into osteoclasts. In human monocytes, cyclic stretch promotes osteoclast differentiation by receptor activator for nuclear factor-κB ligand (RANKL) [32]. On the other hand, in RAW264.7 cells, cyclic stretch inhibits osteoclast differentiation by RANKL [33–35], which suggests that different responses depend on the cell differentiation stage and stretch condition. Compressive stimulation also seems to be involved in osteoclast differentiation. In RAW 264.7 and bone marrow-derived macrophages, continuous compression force promotes osteoclast differentiation, and release from compressive force is involved in the suppression of osteoclast differentiation [36–38]. Stretch and compressive stimulation act on tissue-resident macrophages and bone marrow-derived macrophages in peripheral tissues, such as the periodontal ligament, lung, and bone, and the response by these macrophages depends on the surrounding environment, including the scaffold and type of mechanical stress.

**Table 1:** The effects of mechanical stress on macrophages

Type of cells	Mechanical stress patterns	Functional changes	Reference
Human peripheral blood monocytes	Cyclic stretch 7, 12%	Increase in IL-6, MCP-1, IL-10 mRNA	[68]
Human alveolar macrophages, human monocyte-derived macrophages, human macrophage-like cell line (THP-1)	Cyclic stretch	Increase in IL-8, IL-6, TNF-α protein	[26]
Human macrophage-like cell line (THP-1)	Cyclic stretch + titanium particles	Increase in COX2 mRNA and PGE_2_	[27]
Rat peritoneal macrophages	Static or cyclic stretch	Static stretch induced iNOS and IL-6 mRNA	[28]
Rat alveolar macrophages	Cyclic stretch, 60 cycles/min, 30%	No effect on IL-6 and TNF-α protein	[29]
Mouse macrophage-like cell line (RAW264.7), mouse bone marrow-derived macrophages	Cyclic stretch, 1 Hz	No effect on IL-1β, IL-6, TNF-α, and COX2 mRNA	[30]
Rat alveolar macrophages	Cyclic stretch, 0.5 Hz, 8–20%	Increase in IL-1β protein	[49]
Mouse macrophage-like cell line (J774.1), mouse bone marrow-derived macrophages	Cyclic stretch, 1–30 cycles/min, 5–20%	Decrease in IL-1β protein	[51]
Human macrophage-like cell line (U937)	Cyclic stretch, 0.25 Hz, 10%	Increase in IL-6 protein, esterase, and acidic phosphatase activity	[69]
Human monocyte-derived macrophages	Cyclic stretch (biaxial), 1 Hz, 4%	Increase in MMP-1, MMP3 mRNA	[31]
Human monocyte-derived macrophages and osteoclasts	Cyclic stretch + RANKL	Promotes RANKL-induced osteoclastogenesis	[32]
Mouse macrophage-like cell line (RAW264.7)	Cyclic stretch + RANKL	Inhibits osteoclastogenesis	[33]
Mouse macrophage-like cell line (RAW264.7)	Short-term cyclic stretch + RANKL	Inhibits osteoclastogenesis	[34]
Mouse macrophage-like cell line (RAW264.7)	Cyclic stretch, 1 Hz, 1000 μstrain	Inhibits osteoclastogenesis	[35]
Mouse macrophage-like cell line (RAW264.7)	Compressive force	Promotes osteoclastogenesis	[36]
Mouse bone marrow macrophages	Compressive force	Promotes osteoclastogenesis	[37]
Mouse macrophage-like cell line (RAW264.7)	Release of compressive force	Inhibits osteoclastogenesis	[38]

## Experimental methods of mechanical stimulation to macrophages

**Fig. 1:**
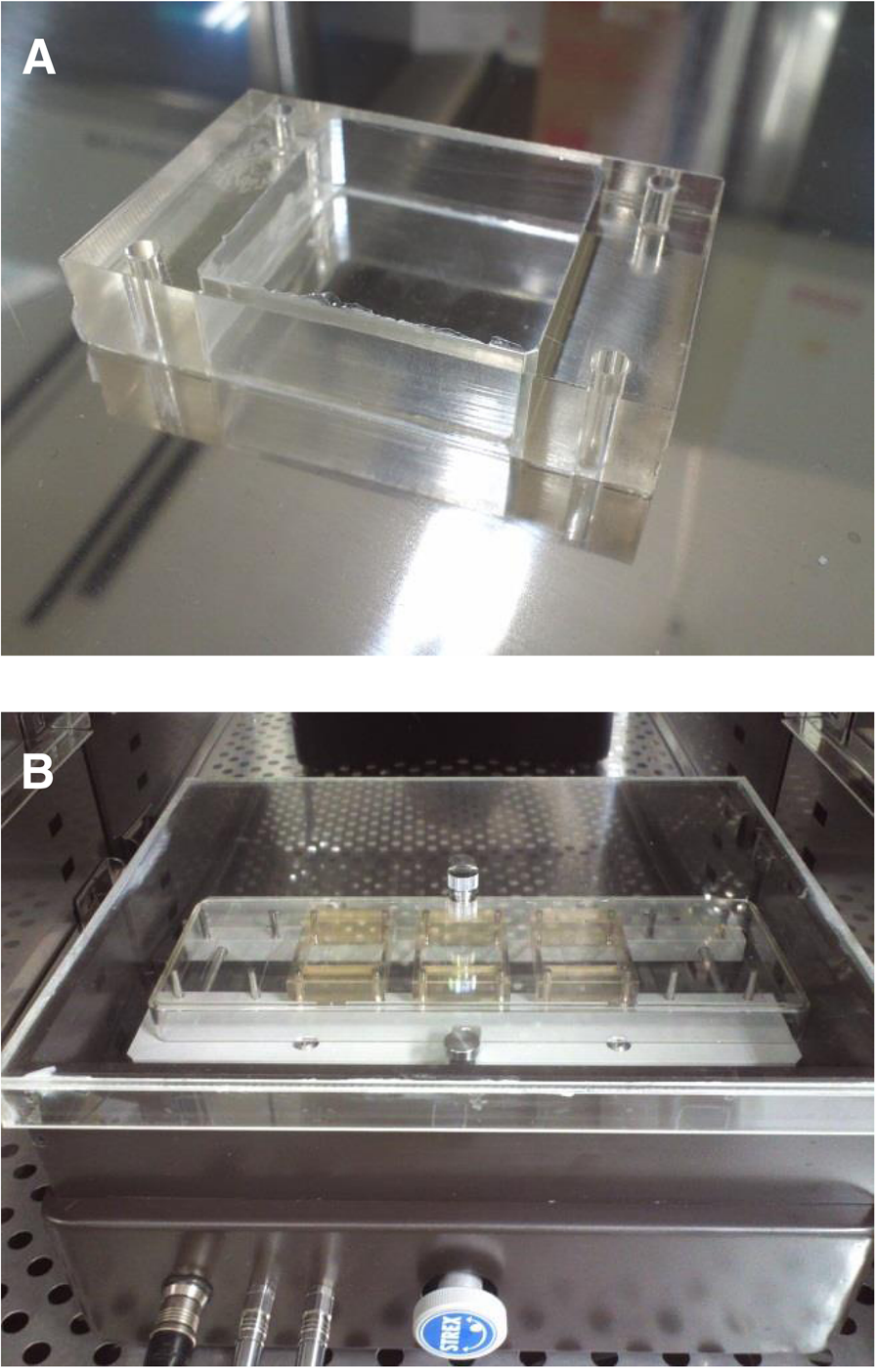
Cell-stretching device. Silicon resin chamber STB-CH-10.0 (**a**). Cell-stretching device STB-140 (**b**)

Some methods have been reported to reproduce mechanical stimulus received by cells in tissue in vitro. In particular, there are many reports on stretch stimulation devices. Devices for verifying the in vitro effect of physical changes of tissues from stretching on macrophages have been used. One cell extension device that is often used is by stretching a silicon resin chamber under negative pressure. Devices that extend the silicon chamber using computer-controlled motors and devices, such as a four-point bending system, are also used. By changing the setting of these devices, it is possible to adjust cyclic and static stimulation, the cell elongation rate, the extension frequency, and the devices can mimic the stimulation that cells receive in the target tissue. In our laboratory, we have investigated the response of cells assuming a periodontal ligament tissue with mechanical stress using a cell-stretching device with a computer-controlled motor for stretching the silicon resin chamber in a controlled (5% CO2) humidified atmosphere (STB-140 STREX cell stretch system (STREX Co., Osaka, Japan)) [39] (Fig. 1). This device can set an extension rate (2, 4, 6, 8, 10, 12, 15, 20%) and an extension frequency (1, 2, 6, 10, 20, 30, 60 cycles/min) to investigate the cellular response to mechanical stress under various conditions.

## NLRP3 inflammasome

IL-1β is secreted from macrophages and promotes the secretion of various inflammatory cytokines during inflammation [40, 41]. The inflammasome, a group of cytosolic protein complexes, strictly controls IL-1β secretion from macrophages [42]. Inflammasomes are a group of cytosolic protein complexes that regulate the activation of caspase-1, convert precursor pro-IL-1β and pro-IL-18 into their mature forms, and consist of cytoplasmic receptors such as a nucleotide-binding oligomerization domain (NOD)-like receptor protein (NLRP), the apoptosis-associated speck-like protein containing a caspase recruitment domain (ASC), and pro-caspase-1 [43]. Inflammasomes are classified into several types depending on the activated intracellular receptors [44]. The inflammasome NLRP3 reacts to extracellular adenosine triphosphate (ATP), β-amyloid, and cholesterol [45–48]. The activation mechanism of the inflammasome begins with the induction of pro-IL-1β and constitutive inflammasome molecules (signal 1). Macrophages recognize bacterial cell components, such as lipopolysaccharide (LPS) or inflammatory cytokines, such as TNF-α and IL-1β, which in turn induce the expression of pro-IL-1β and constitutive inflammasome molecules via nuclear factor-kappa B (NF-κB) signaling. Next, NLRP3 inflammasome components are assembled after sensing danger signals, such as pathogen-associated molecular patterns (PAMPs) and damage-related molecular patterns (DAMPs), and activate caspase-1, which processes pro-IL-1β into mature IL-1β (signal 2).

## Relationship between mechanical stress and the NLRP3 inflammasome

**Fig. 2:**
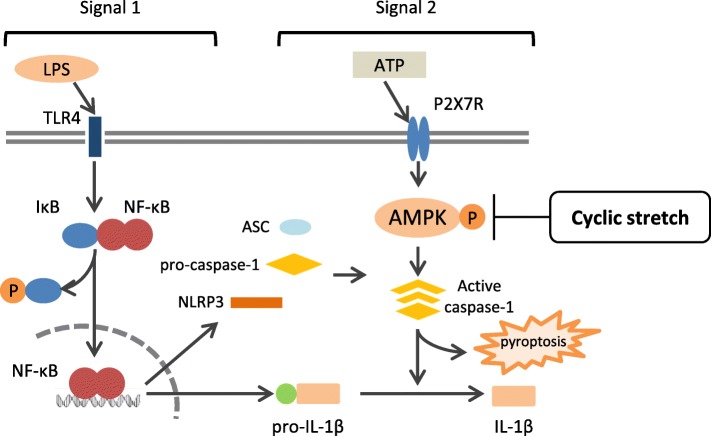
NLRP3 inflammasome pathways and putative mechanism by which cyclic stretch negatively regulates IL-1β secretion in murine macrophages. Treatment with LPS activates NF-κB signaling via toll-like receptor (TLR) 4 (signal 1) and induces the expression of NLRP3 and pro-IL-1β. Extracellular ATP activates inflammasomes via P2X7 receptors (signal 2) and induces the activation of caspase-1, which leads to the secretion of IL-1β and pyroptosis. Cyclic stretch does not interfere with NF-κB signaling (signal 1), but inhibits the activation of caspase-1 (signal 2) by attenuating the AMP kinase pathway

There are a few reports on the relationship between mechanical stress and the NLRP3 inflammasome. Wu et al. reported that cyclic stretch activates the NLRP3 inflammasome via mitochondrial ROS production in tissue-resident mouse alveolar macrophages and suggested that this mechanism may be related to lung inflammation induced by mechanical ventilation [49]. This report indicates that mechanical stress may be a risk factor of NLRP3 inflammasome activation. While Stojadinovic et al. reported that sustained compressive force to epidermal tissue resulted in enhanced protein expression of NLRP3 and caspase-1, but decreased the expression of IL-1β [50]. Therefore, the relationship between mechanical stress and inflammasome signaling is still unclear. Recently, we found that cyclic stretch suppresses the NLRP3 inflammasome in macrophages [51] and will introduce our new findings in the latter part of this paper (Fig. 2).

## Suppression of IL-1β secretion by cyclic stretch in macrophages

**Fig. 3:**
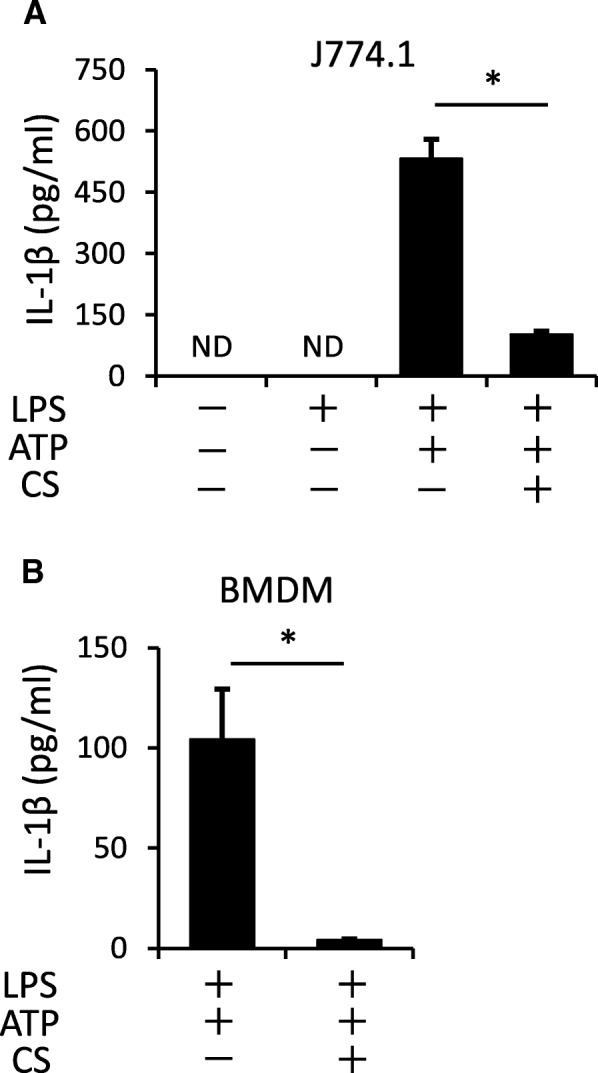
Cyclic stretch inhibits ATP-stimulated IL-1β secretion in LPS-primed macrophages. The murine macrophage cell line J774.1 (**a**) and mouse bone marrow-derived macrophages (BMDM) (**b**) was primed with 100 ng/mL of *E. coli* LPS for 4 h followed by stimulation with 1 mM ATP for 2 h in the continuous presence of LPS. Cells were exposed to cyclic stretch of 20% elongation at a frequency of 10 cycles/min for the first 2 h after the addition of LPS. Significance is indicated (*P < 0.05 significantly different from the positive control). CS, cyclic stretch. ND, not detected

Extracellular ATP released from injured cells or bacteria is recognized by the macrophage P2X7 receptor, which causes a loss of potassium ions and activates the NLRP3 inflammasome [52, 53]. ATP triggers IL-1β secretion in LPS-primed J774.1 mouse macrophages (Fig. 3a), but excessive inflammation by the NLRP3 inflammasome may disrupt tissue homeostasis [42]. We examined the relationship between mechanical stress and the NLRP3 inflammasome using a cyclic stretch system. LPS-primed J774.1 cells and mouse bone marrow-derived macrophages were exposed to a cyclic stretch of 20% elongation at a frequency of 10 cycles/min, which markedly suppressed IL-1β secretion (Fig. 3), suggesting that cyclic stretch inhibits the NLRP3 inflammasome signaling pathway.

## Cyclic stretch does not inhibit the NF-κB pathway in macrophages

**Fig. 4:**
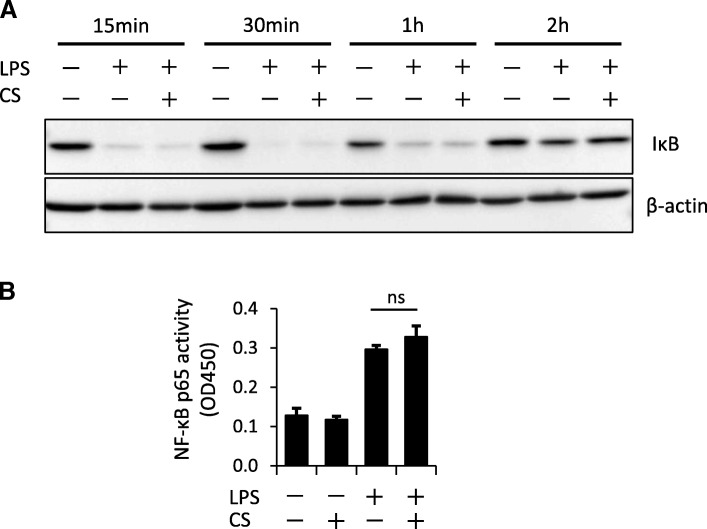
Cyclic stretch does not alter the LPS-induced NF-κB signaling pathway. **a** J774.1 cells were exposed to cyclic stretch of 20% elongation at a frequency of 10 cycles/min with 100 ng/mL LPS for the indicated times. Cell lysates were analyzed by western blotting with anti-IκB-α. An antibody against β-actin was used as a control. **b** J774.1 cells were exposed to cyclic stretch of 20% elongation at a frequency of 10 cycles/min for the first 2 h during treatment with 100 ng/mL LPS for 4 h. Nuclear proteins were extracted from cells and an NF-κB ELISA assay was performed. CS, cyclic stretch. ns, not significant

Expression of NLRP3 inflammasome-related molecules, such as NLRP3 and pro-IL-1β, is required for the activation of the NLRP3 inflammasome. These molecules are induced by the activation of the NF-kB pathway by bacterial components such as LPS (signal 1) [54]. We investigated whether cyclic stretch inhibits the NF-kB pathway. Inhibitor of κB (IκB), which binds to the NF-κB complex in the cytoplasm at steady state, is phosphorylated by inhibitor of κB kinase (IKK) and degraded via a ubiquitin-proteasome degradation system when a stimulus, such as LPS, is added to the cells [55]. Figure 4a shows that cyclic stretch had no effect on LPS-induced IκB time-dependent degradation and re-expression. Liberated NF-κB translocates to the nucleus and binds to the promoters of NF-κB target genes including pro-inflammatory cytokines and NLRP3 inflammasome-related genes [56, 57]. We also examined whether cyclic stretch inhibits the transcriptional activity of NF-κB in the nucleus. Proteins from the nucleus of J774.1 macrophages primed by LPS were extracted and examined using an NF-κB p65 DNA-binding ELISA method. As the result, cyclic stretch did not significantly affect LPS-induced NF-κB p65-binding activity (Fig. 4b), which suggests that suppression of IL-1β secretion by cyclic stretch is independent of NF-κB signaling (signal 1).

## Cyclic stretch suppresses caspase-1 activation in macrophages

**Fig. 5:**
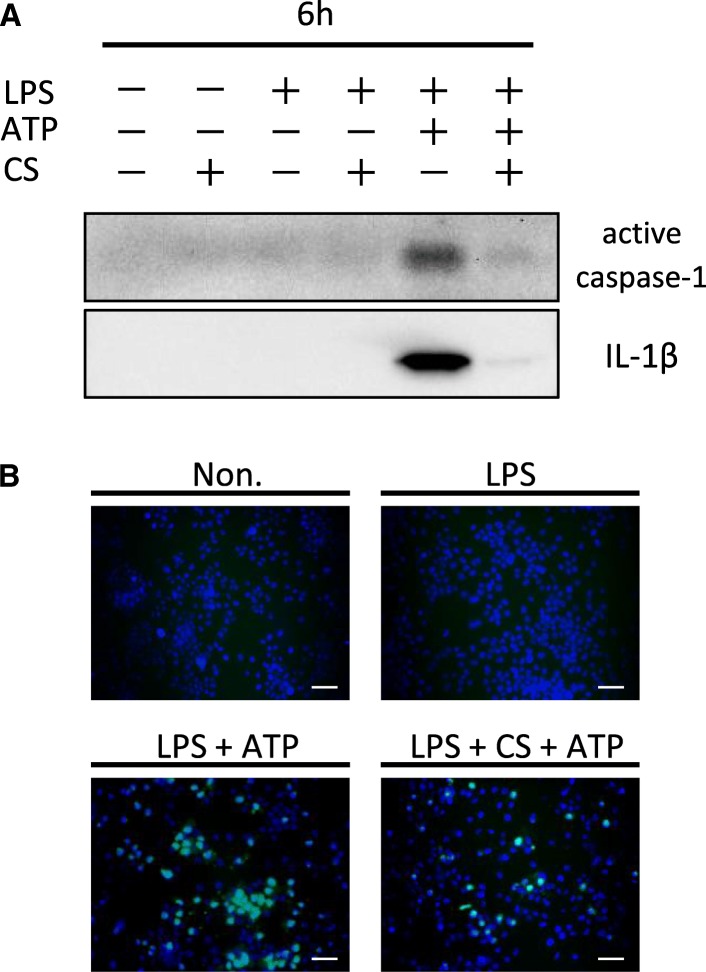
Cyclic stretch inhibits LPS/ATP-induced activation of caspase-1. J774.1 cells were exposed to cyclic stretch of 20% elongation at a frequency of 10 cycles/min for the first 2 h during treatment with 100 ng/mL LPS for 4 h followed by stimulation with ATP for 2 h in the continuous presence of LPS. **a** Concentrated supernatants were analyzed by western blotting with specific antibodies to caspase-1 and IL-1β. **b** Cells were labeled with a FLICA probe conjugated with FAM (green) and nuclei were visualized by staining with Hoechst 33342 (blue) (magnification, × 200; scale bars are 50 μm). The negative control (Non.) was not treated with LPS, ATP, or cyclic stretch. CS, cyclic stretch

The NLRP3 inflammasome signal 2 consists of a signal cascade that begins with the recognition of danger signals [45]. Activation of NLRP3 inflammation is induced by potassium ion efflux via ATP binding to P2X7 cell membrane receptors and reactive oxygen species (ROS) production in the cytoplasm, which in turn converts pro-caspase-1 to active caspase-1 [52]. Therefore, we examined the effect of cyclic stretch on the activation of caspase-1 using western blotting and a FLICA probe-conjugated FAM, which specifically detects active caspase-1 in the cytoplasm. Expression of released activated caspase-1 by inflammasome activation and the number of cells with the active form of caspase-1 in the cytoplasm were suppressed by cyclic stretch in ATP-stimulated LPS-primed J774.1 cells (Fig. 5).

## AMPK controls the NLRP3 inflammasome

Adenosine monophosphate-activated protein kinase (AMPK) signaling is a key regulator of cellular energy homeostasis. This signaling pathway mainly functions as the cell’s energy sensor and controls various functions such as metabolic regulation, cytoskeleton regulation, and the inflammatory response [58, 59]. AMPK regulates the activation of the NLRP3 inflammasome with aging, and activation of AMPK signaling may be a therapeutic target for age-related diseases. Autophagy promotion, mitochondrial homeostasis, endoplasmic reticulum stress regulation, and SIRT1 activation by AMPK suppresses NLRP3 inflammasome activation [60]. Activation of the AMPK signaling pathway is involved in NLRP3 inflammasome suppression, but some reports suggest that inhibition of AMPK activation suppresses NLRP3 inflammasome activation. Phosphorylation of AMPK activates the NLRP3 inflammasome and promotes IL-1β secretion and pyroptosis, and suppression of AMPK phosphorylation inhibits the NLRP3 inflammasome [61]. Piperine (an alkaloid contained in black pepper) inhibits AMPK phosphorylation, which accompanies extracellular ATP stimulation in mouse macrophages J774A.1 and human proximal tubular cell line HK-2 cells, and suppresses NLRP3 inflammasome activation [62, 63]. In addition, in mice placed on a ketogenic diet, reduction of AMPK phosphorylation in the retina and concomitant suppression of NLRP3 inflammasome are observed [64]. We found that cyclic stretch suppresses macrophage NLRP3 inflammasome via inhibition of ATP-triggered AMPK phosphorylation. AMPK phosphorylation in LPS-primed macrophages was significantly enhanced by adding extracellular ATP, but cyclic stretch suppressed this phosphorylation, which indicates that AMPK signaling participates in inflammasome signaling and mechanical stress. Phosphorylation of AMPK is regulated by cyclic stretch [65–67], and mechanical stress regulates AMPK signaling in inflamed and normal cells. Therefore, mechanical stress is a factor that regulates NLRP3 inflammasome via AMPK signaling.

## Conclusion

Although the role of mechanical stress in the inflammatory response is still controversial, our findings provide insight into the maintenance of homeostasis through the prevention of excessive inflammasome activation.

## Abbreviations

AMPK: Adenosine monophosphate-activated protein kinase
ASC: Apoptosis-associated speck-like protein containing a caspase recruitment domain
ATP: Adenosine triphosphate
DAMPs: Damage/danger-associated molecular patterns
FAK: Focal adhesion kinase
IKK: Inhibitor of kappa B kinase
IL: Interleukin
IκB: Inhibitor of kappa B
LDH: Lactate dehydrogenase
LPS: Lipopolysaccharide
MAP kinase: Mitogen-activated protein kinase
NF-κB: Nuclear factor-kappa B
NLRP: Nucleotide-binding oligomerization domain-like receptor protein
NLRP3: NLR family, pyrin domain containing 3
PAMPs: Pathogen-associated molecular patterns
ROS: Reactive oxygen species
TNF-α: Tumor necrosis factor-α
